# Establishment and Characterization of Primary Glioblastoma Cell Lines from Fresh and Frozen Material: A Detailed Comparison

**DOI:** 10.1371/journal.pone.0071070

**Published:** 2013-08-07

**Authors:** Christina Susanne Mullins, Björn Schneider, Florian Stockhammer, Mathias Krohn, Carl Friedrich Classen, Michael Linnebacher

**Affiliations:** 1 Pediatrics Department, University Hospital Rostock, Rostock, Germany; 2 Department of Surgery, University Hospital Rostock, Rostock, Germany; 3 Department of Pathology, University Hospital Rostock, Rostock, Germany; 4 Department of Neurosurgery, University Hospital Rostock, Rostock, Germany; University of Michigan School of Medicine, United States of America

## Abstract

**Background:**

Development of clinically relevant tumor model systems for glioblastoma multiforme (GBM) is important for advancement of basic and translational biology. High molecular heterogeneity of GBM tumors is well recognized, forming the rationale for molecular tests required before administration of several of the novel therapeutics rapidly entering the clinics. One model that has gained wide acceptance is the primary cell culture model. The laborious and time consuming process is rewarded with a relative high success rate (about 60%). We here describe and evaluate a very simple cryopreservation procedure for GBM tissue prior to model establishment that will considerably reduce the logistic complexity.

**Methods:**

Twenty-seven GBM samples collected ad hoc were prepared for primary cell culture freshly from surgery (#1) and after cryopreservation (#2).

**Results:**

Take rates after cryopreservation (59%) were as satisfactory as from fresh tissue (63%; p = 1.000). We did not observe any relevant molecular or phenotypic differences between cell lines established from fresh or vitally frozen tissue. Further, sensitivity both towards standard chemotherapeutic agents (Temozolomide, BCNU and Vincristine) and novel agents like the receptor tyrosine kinase inhibitor Imatinib did not differ.

**Conclusions:**

Our simple cryopreservation procedure facilitates collection, long-time storage and propagation (modeling) of clinical GBM specimens (potentially also from distant centers) for basic research, (pre-) clinical studies of novel therapies and individual response prediction.

## Introduction

Glioblastoma multiforme (GBM) is the most aggressive primary brain tumor in adults with currently no cure. The tumors can appear *de novo* without a previously diagnosed tumor (primary GBM) or through progression from low-grade astrocytic or oligodendrocytic tumors (secondary GBM) [1]. Although clinically indistinguishable, primary and secondary GBM display specific molecular features. Primary GBM often show an amplification and/or mutation of the endothelial growth factor receptor (EGFR). The most common mutation is the variant III (EGFRvIII), a mutation leading to a weak but constitutively active receptor signaling [2], [3]. Secondary GBM are associated with mutations of the tumor suppressor gene TP53 as well as mutations in the genes IDH 1 and 2 [4], [5]. The main purpose for generating *in vitro* models of brain tumors is to identify mechanisms contributing to oncogenesis or tumor maintenance starting with analysis of distinct molecular patterns and to define and evaluate potential therapeutic strategies. “Targeted” therapies “only” need molecular testing but for functional analyses such as response prediction vital and proliferating malignant cells are indispensable [6], [7]. On top, they provide a nearly unlimited supply of material for all sorts of studies. There are two types of patient individual tumor models: *in vitro* (primary cell cultures) and *in vivo* (patient derived xenografts in immunodeficient animals) [8], [9]. These models should be passaged as little as possible preventing epigenetic or genetic alterations and thus keeping them close to the original tumor [10], [11]. Moreover, it is important to establish models from individual tumors in order to cover a broad spectrum and to ensure that the genetic heterogeneity of a given tumor entity is fully represented. These individual models allow the most accurate response and resistance prediction outside the patient. The high precision of therapy prediction with such individual models in carcinomas could be demonstrated by Voskoglou-Nomikos and colleagues as well as by Fiebig and co-workers with 90% and even 97% accuracy rates for prediction of response and resistance, respectively [12], [13].

Being up-to-date is especially important in the field of clinical research. And one of the big buzzwords of the present is the term “individualized therapy”! Patient-individual tumor models clearly would simplify taking the approach of individualized therapy to the next level. It will not only be possible to define responders and non-responders for individual therapy regimens and combinational treatment schedules but also dissecting the relevant causes here for. In line with these opportunities is also the chance to perform decentralized sample collection (which is relatively easy to standardize) along with more centralized model establishment units. In short, the present technical study aims at profoundly simplifying the complex logistics of establishing individual GBM models and paving the way for a multitude of possible preclinical and clinical applications.

## Materials and Methods

### Patient Cohort

Between august 2009 and august 2011, 26 clinical samples from patients with WHO grade IV GBM and one patient with a relapsed Astrocytoma, WHO grade III (Table 1) were collected from the Neurosurgery department at the University medicine Rostock. Prior informed consent was obtained in written form from all patients, and all procedures were approved by the institutions’ Ethics Committee: “Ethikkommission an der Medizinischen Fakultät der Universität Rostock” (reference number: A 2009/34) in accordance with general accepted guidelines for the use of human material.

**Table 1:** Clinical information.

Tumor ID	Age	Gender	Localization	Comment	Survival (months)
HROG02	68	M	R; parietooccipital		† 7
HROG04	53	F	R; frontal	relapse	†13
HROG05	60	F	L; temporal	relapse	† 3
HROG06	53	M	L; frontal		† 8
HROG07	55	M	R; temporoparietal	relapse	† 6
HROG10	74	M	R; temporal		† 7
HROG11	54	F	R; frontal		30
HROG12	64	M	R; frontoparietal		† 5
HROG13	77	F	R; temporal		† 8
HROG15	56	M	R; parietal		23
HROG16	53	M	R; parietal		† 26
HROG17	70	M	L; parietooccipital	relapse	† 3
HROG19	69	M	L; temporoparietal	relapse	† 15
HROG21	44	M	R; parietal	secondary GBM	21
HROG22	66	M	L; temporal	relapse	† 4
HROG23	60	F	L; parietal	relapse	20
HROG24	73	F	L; occipital		† 10
HROG25	77	F	L; temporal	relapse	† 3
HROG26	63	M	R; parietal	relapsed Astrocytoma	† 8
HROG31	59	F	R; occipitotemporal		21
HROG32	76	F	R; temporal		22
HROG33	46	F	L; occipitotemporal		† 13
HROG34	69	F	L; frontal		† 5
HROG36	80	F	R; parietal		† 5
HROG38	49	F	R; parietooccipital		19
HROG41	71	M	L; frontal		† 2
HROG42	70	F	L; frontal		16

Relevant clinical patient data concerning age (at time point of surgery), gender (M = male; F = female), tumor localization, further information (if provided) and survival in months († = patient died; bold = patient still alive on January 25^th^ 2013) are summarized.

### Tumor Specimen Collection and Cryopreservation

Resection specimens of GBM tumors (n = 27) were received sterile and freshly from surgery. Tumor tissue samples were snap frozen in liquid nitrogen and stored in the gas phase above liquid nitrogen. Additionally, tumor tissue cubes (3×3×3 mm) were frozen vitally. For this procedure, tumor pieces were cut with a sterile scalpel blade, and 4 tumor pieces were transferred into one sterile cryo-tube in 1.5 ml freezing medium (fetal calf serum containing 10% DMSO), sealed in a freezing container (Nalgene, Rochester, USA), and placed immediately at −80°C. Until unthawing, tubes were kept at −80°C (for a maximum of 6 weeks) or, after overnight cooling, transferred into a nitrogen tank (for longer storage periods). For subsequent culture procedures, cryopreserved tumor pieces were thawed at 37°C.

### Tissue Culture and Cell Line Establishment

Tumor tissue (fresh or vitally frozen and then thawed) was minced (by crossed scalpels) in DMEM/Ham’s F12 cell culture media supplemented with 10% FCS, 2 mM L-glutamine and penicillin-streptomycin and passed through a cell strainer (100 µm; Becton-Dickinson-Falcon, Heidelberg, Germany) to obtain a single cell suspension. Cells were washed with PBS and seeded in 6 well plates coated with collagen. Outgrowing cells were detached with trypsin and transferred to T25 cell culture flasks. Cells passaged 2–3 times in this manner were transferred to T175 culture flasks and expanded for subsequent analyses. All cell culture plastics were from Greiner Bio one, Frickenhausen, Germany and cell culture media and supplements were purchased from PAA, Cölbe, Germany.

### Phenotypic Characterization (Microphotography)

Cells were cultured in T25 flasks to a confluence of 60–80% and photographed using the AxioVision 4.8.2 software (Carl Zeiss, Jena, Germany). Photographs were edited with Photoshop CS3 (Adobe, München, Germany).

### Growth Kinetics

Cells (5×10^5^ cells) were plated in 5 ml media in quintuplicate T25 culture flasks per cell line and allowed to attach for 48 h; vital cells were assessed by trypan blue staining and one flask was counted every 24 h for five consecutive days using a Neubauer chamber.

### Flow Cytometry

The expression of neuronal markers was assessed by flow cytometry. Cells were harvested, counted and 5×10^5^ cells were stained with 1 µg of mouse anti-human antibodies labeled with Alexa Fluor 488 directed against either GFAP (clone GA5; eBioscience, Frankfurt, Germany) or nestin (clone 10C2; eBioscience). Cells stained with an irrelevant antibody of the same isotype served as negative controls. Similarly, 5×10^5^ cells were stained with 0.06 µg mouse anti-human antibody directed against vimentin (clone V9; Abcam, Cambridge, UK) and stained with a secondary anti-mouse FITC-labeled antibody (DakoCytomation, Glostrup, Denmark). For the latter staining, cells handled the same way with no primary antibody served as negative control. All incubations were performed in PBS on ice for 30 min.

### Molecular Characterization

Genomic DNA (gDNA) from snap frozen tumor tissue and cell culture cell pellets (3×10^6^ cells) was isolated using the Wizard Genomic DNA Purification Kit (Promega, Mannheim, Germany) according to the manufacturer’s instructions. Concentration of isolated gDNA was determined with the NanoDrop1000 (Thermo-Scientific, Wilmington, USA).

#### EGFR copy number analysis

For determination of EGFR copy number, quantitative PCR was performed. 30 ng gDNA were used as template. The run was performed on a StepOne Realtime PCR system (Applied Biosystems, Darmstadt, Germany) using Fast SYBR Green Mastermix (Applied Biosystems). Commercial normal human gDNA (Promega) was used as calibrator and the repetitive element LINE1 as endogenous control. The calculation of the EGFR copy number was performed using the ΔΔCt-algorithm. All reactions were performed in triplicates. Primers used for EGFR copy number analysis were: EGFR-forward: 5′-TCCCATGATGATCTGTCCCTCACA-3′; EGFR-reverse: 5′-CAGGAAAATGCTGGCTGACCTAAG-3′; LINE1-forward: 5′-TGCTTTGAATGCGTCCCAGAG-3′; LINE1-reverse: 5′-AAAGCCGCTCAACTACATGG-3′.

#### MGMT promoter methylation analysis

For analyzing the MGMT promoter concerning methylation the MethyLight method was applied [14]. Briefly, gDNA was subject to bisulfite conversion using the Epitect Bisulfite Kit (Qiagen, Hilden, Germany) according to the manufacturer’s recommendations. A primer/probe combination specific for methylated MGMT promoter sequence was used (forward: 5′-GCGTTTCGACGTTCGTAGGT-3′; reverse: 5′-CACTCTTCCGAAAACGAAACG-3′; probe: 5′-6FAM-CGCAAACGATACGCACCGCGA-TMR-3′), with SensiFast Probe Kit (Bioline, Luckenwalde, Germany). CpG Methylase (SssI) treated DNA served as calibrator, as it is considered as fully methylated. The collagenase gene 2A1 (COL2A1), was used as endogenous control (forward: 5′-TCTAACAATTATAAACTCCAACCACCAA-3′; reverse: 5′-GGGAAGATGGGATAGAAGGGAATAT-3′; probe: 5′-6FAM-CCTTCATTCTAACCCAATACCTATCCCACCTCTAAA-TMR-3′). The percentage of methylated reference (PMR) value was calculated by dividing the MGMT/COL2A1 ratio of the sample by the MGMT/COL2A1 ratio of the SssI-treated DNA, and multiplying by 100. Samples with a PMR value >4 were considered as methylated [14]. All reactions were performed in triplicates.

#### Mutation analyses

Samples underwent analyses for the following loci: IDH 1 R132 (exon 4), IDH 2 R172 (exon 4), B-Raf V600 (exon 15), K-Ras G12, G13 (exon 2) and Q61 (exon 3) and TP53 exons 5 to 8. The desired genomic regions were amplified by PCR using specific primers (see Table 2). The PCR was performed using MyTaqHS polymerase (Bioline) according to the manufacturer’s recommendations. The PCR reaction was controlled by agarose gel electrophoresis and 15 µl of the products were purified using 3 units of FAST AP™ Alkaline Phosphatase (Fermentas, St. Leon-Rot, Germany) and 30U of Exonuclease I (Fermentas) by incubation at 37°C for 15 min and subsequent heat inactivation at 85°C for 15 min.

**Table 2:** Primers used for mutation analyses.

Target	Forward Primer	Reverse Primer
IDH 1 exon 4	5′-GCACGGTCTTCAGAGAAGCC-3′	5′-CACATTATTGCCAACATGAC-3′
IDH 2 exon 4	5′-GCCCACACATTTGCACTCTA-3′	5′-CAGAGACAAGAGGATGGCTAGG-3′
B-Raf exon 15	5′-TCATAATGCTTGCTCTGATAGGA-3′	5′-CTTTCTAGTAACTCAGCAGC-3′
K-Ras exon 2	5′-GTACTGGTGGAGTATTTGATAGTGTATTAA-3′	5′-TCAAAGAATGGTCCTGCACC-3′
K-Ras exon 3	5′-CTTTGGAGCAGGAACAATGTCT-3′	5′-TACACAAAGAAAGCCCTCCCC-3′
TP53 exon 5	5′-(GC40)TTCCTCTTCCTACAGTACTC-3′	5′-CTGGGCAACCAGCCCTGTCGT-3′
TP53 exon 6	5′-(GC40)GACGACAGGGCTGGTTGCCCA-3′	5′-AGTTGCAAACCAGACCTCAG-3′
TP53 exon 7	5′-(GC40)TCTCCTAGGTTGGCTCT-3′	5′-GCAAGTGGCTCCTGACCTGG-3′
TP53 exon 8	5′-CCTATCCTGAGTAGTGGTAATC-3′	5′-(GC40)CCGCTTCTTGTCCTGCTTGCTT-3′

One µl of the PCR product was used as template for Sanger sequencing using BigDye® Terminator v1.1 Cycle Sequencing Kit (Applied Biosystems) and the primers used for PCR according to the manufacturer’s protocol. The sequencing products were purified using the BigDye XTerminator® Purification Kit (Applied Biosystems). The sequence was analyzed using the 3500 genetic analyzer system (Applied Biosystems) and the SeqScape® Software v2.7 (Applied Biosystems).

### Drug Response

Cells (5×10^3^ cells) were plated in 150 µl media (as above) per well in triplicate in 96 well flat bottom culture plates and allowed to attach for 24 h. The following concentration ranges of drugs were tested (given are final concentrations in the experimental wells): (1) 2 mM–128 nM Temozolomide (Sigma Aldrich, Schnelldorf, Germany), (2) 500 µM–32 nM BCNU (Bristol-Myers Squibb, New York, USA), (3) 244 nM–300 pM Vincristin and (4) 250 µM–60 nM Imatinib (Novartis, Basel, Switzerland). Equal volumes DMSO (for cells treated with Temozolomide and BCNU) were added to cells serving as live control. Cells were incubated with the substances for 72 h, and media were replaced together with substances in the same concentrations as before. After another 72 h incubation period cells serving as dead control were incubated with 70% ethanol for 30 min and viability was assessed by using the viability dye calcein AM (eBioscience, Frankfurt, Germany) in a final concentration of 0.7 µM in fresh medium:PBS (2∶1). Cells were incubated at 37°C in the dark for 20 min, fluorescence intensity was assessed using the microplate reader Infinite M200 (Tecan, Mennedorf, Switzerland) with 485 nm excitation, 535 nm emission and a constant gain of 160. Values were normalized (1 = value live control; 0 = value dead control).

### Statistics

All statistics (T-test, Fisher’s Exact test, Mann-Whitney U test and IC_50_-values) were done using the statistics program SigmaStat3.5 and SigmaPlot 10.0.

## Results

### Success Rates

We assessed attachment and outgrowth rates of 26 consecutive WHO grade IV GBM tumor samples and one relapsed Astrocytoma, when prepared fresh directly after resection (culture #1) or after vital storage for varying periods of time in liquid nitrogen (culture #2). After fresh preparation, cells attached in 85% (24/27) of the cases and after vital freezing before preparation, attachment of cells occurred in 78% (21/27; p = 1.000). Establishment of stable outgrowing cell lines was successful in 63% (17/27) of freshly prepared material and in 59% (16/27; p = 1.000) of vitally frozen material prior to preparation (summarized in Table 3). The five most rapidly and stable outgrowing pairs of cell cultures were subsequently characterized in detail. In the following, stable outgrowing cultures (could be passaged >40 times) are termed cell lines. Cell lines derived from fresh material were marked with the suffix #1 and cell lines from vitally frozen material with the suffix #2.

**Table 3:** Success rates of *in vitro* models.

cell line	success		cell line	success		cell line	success	
	#1	#2		#1	#2		#1	#2
HROG02	✓	✓	HROG15	✓	✓	HROG26		
HROG04	✓	✓	HROG16			HROG31		
HROG05	✓	✓	HROG17	✓	✓	HROG32		✓
HROG06	✓	✓	HROG19			HROG33	✓	✓
HROG07	✓	✓	HROG21	✓	✓	HROG34		✓
HROG10	✓	✓	HROG22		✓	HROG36	✓	✓
HROG11	✓	✓	HROG23	✓		HROG38	✓	
HROG12			HROG24	✓		HROG41	✓	
HROG13	✓	✓	HROG25			HROG42		

A comparative overview on the success of cell line establishment from the fresh and vitally frozen tumor material; successful cell line establishment is indicated by a check mark.

### Morphology and Growth Kinetics

**Figure 1:**
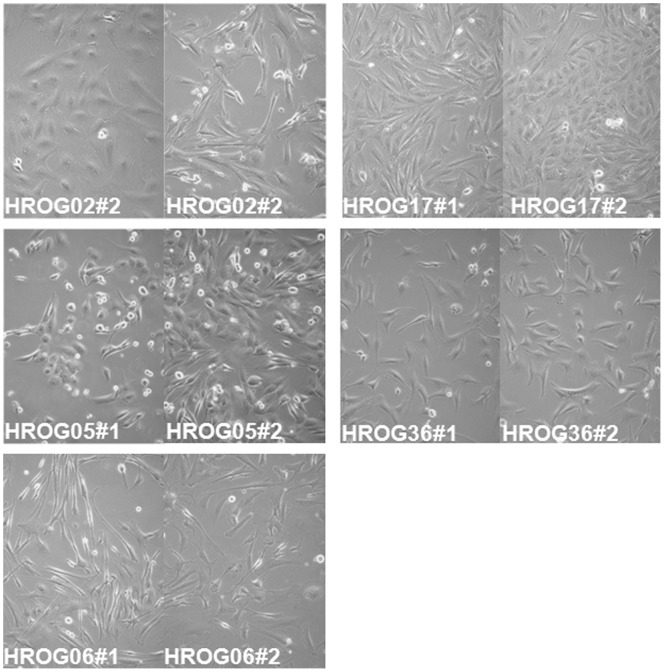
Cell line morphology. Phenotypes of the cell lines captured by microphotography are displayed pairwise.

**Figure 2:**
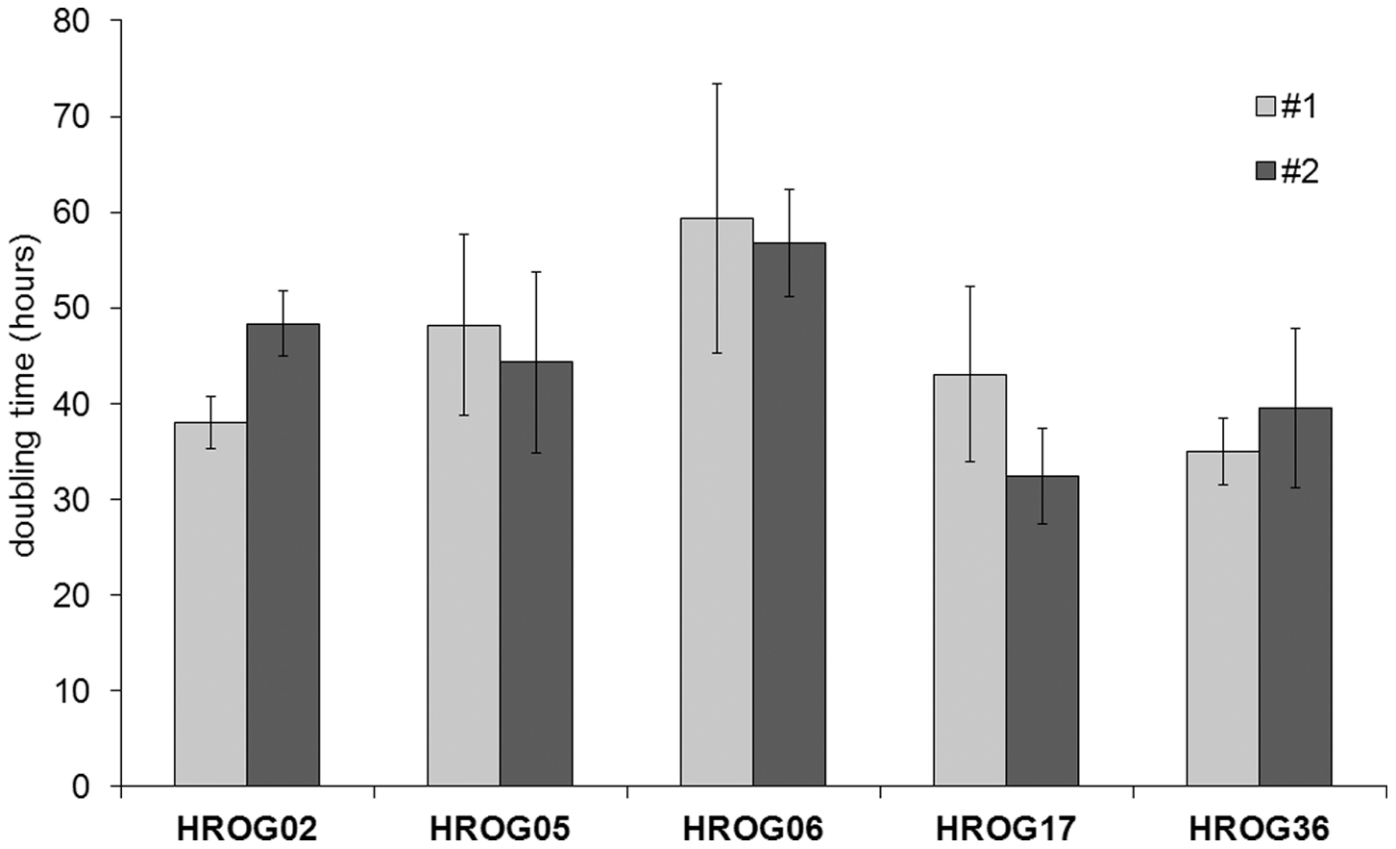
Doubling times. Mean doubling times for the cell lines are displayed placing the pairs side by side. The doubling time for each cell line was determined three times; the mean doubling time (in h) and standard deviation were calculated and depicted in a bar chart.

In a first step, the cell lines were micro-photographed to compare the morphology of the cell line pairs. In Figure 1, the morphology of the newly established tumor cell lines is depicted, showing the pairs side by side for a direct comparison. Furthermore, doubling times of the cell lines were assessed and are presented pair wise in Figure 2. In all cases the pairs showed high similarity in regard to their morphology and doubling times but differences between the individual cell lines were obvious. Morphologically all cell lines show a fibroblast-like phenotype, and no differences between freshly cultivated and cell lines from previously frozen tumors became apparent. Doubling times ranged from 35/40 h (#1/#2) for HROG36 to 59/57 h for HROG06. HROG17 having doubling times of 43/32 h, followed by HROG02 with 36/54 h and HROG05 with 48/44 h. All cell lines/pairs expressed the neuronal markers GFAP, nestin and vimentin as assessed by flow cytometry (see Figure S1).

### Molecular Data

Molecular markers relevant for GBM such as the methylation status of the MGMT promoter, the amplification rate of EGFR, as well as mutation status of the genes IDH 1 and 2, TP53, K-Ras and B-Raf were assessed for the cell line pairs in comparison to the original tumor material (Table 4). The methylation status of the MGMT promoter was consistent between original tumor and cell line pairs.

**Table 4:** Molecular data.

	HROG02			HROG05			HROG06			HROG17			HROG36		
	tumor	#1	#2	tumor	#1	#2	tumor	#1	#2	tumor	#1	#2	tumor	#1	#2
**MGMT-**	25^1^	39	47	35	13	58	0	0	0	13	6	4	0	0	0
**promoter**	M^2^	M	M	M	M	M	U	U	U	M	M	M	U	U	U
**EGFR ^3^**	3	1	2	82	2	2	82	3	2	4	1	1	1	1	1
**TP53**	**R248Q**	**R248Q**	**R248Q**	wt	wt	wt	**R273H** R306*	**R273H** R306*	**R273H** R306*	wt	wt	wt	wt	wt	wt
**IDH 1 & 2**	wt	wt	wt	wt	wt	wt	wt	wt	wt	wt	wt	wt	wt	wt	wt
**K-Ras**	wt	wt	wt	**G12D**	**G12D**	**G12D**	wt	wt	wt	wt	wt	wt	wt	wt	wt
**B-Raf**	wt	wt	wt	Wt	wt	wt	wt	wt	wt	wt	wt	wt	wt	wt	wt

Relevant molecular features of the original tumor in comparison to the cell line pairs are displayed. The methylation status of the MGMT promoter is given as PMR value and marked with an M = methylated or U = unmethylated. The gene amplification rate of the EGFR is given as a multiple of the normal diploid status ( = 1). Mutation status of the genes TP53, IDH 1 and 2, K-Ras and B-Raf are termed wt ( = wildtype) if no mutations were detected. Mutations are indicated by the position with the wt amino acid in front and the amino acid resulting from the mutation behind. ^1^PMR value, ^2^ methylation status, ^3^ amplification rate.

The amplification rate of the EGFR differed in four (HROG02, HROG05, HROG06 and HROG17) out of five cases when comparing the status of the original tumor to the cell lines. No differences were, however, observed between the cell line pairs.

Of note, all mutations detected in the original tumors were maintained in the cell lines. HROG02 and HROG06 show a mutation in the TP53 gene and HROG05 has a mutation in the K-Ras gene. No mutations were detected in HROG17 or HROG36 and similarly, we did not observe any hot spot mutations in the genes IDH 1 and 2 or B-Raf.

### Drug Response

For functional comparison of the cell line pairs, the sensitivity of each cell line (pair) towards a panel of therapeutic agents commonly used for GBM treatment was assessed (summarized in Table 5, and detailed information can be depicted from Figure S2). As expected, the response to different drugs varied within a given cell line. Similarly, the response to one agent varied between the different cell lines. Notably, no severe differences in regard to sensitivity to one agent were observed when comparing the cell lines in matched pairs. There was only one exception from this rule. For HROG36 minor dissimilarities were observed with regard to the substance Vincristine. The IC_50_ values of HROG36#1 and HROG36#2 are 79 nM and 42 nM respectively; but HROG36#1 plateaus at about 60% of dead cells. However, this was statistically not significant (p = 1.000).

**Table 5:** IC_50_ values.

cell line	TMZ (IC_50_) [µM]		BCNU (IC_50_) [µM]		Vincristine (IC_50_) [nM]		Imatinib (IC_50_) [µM]	
	#1	#2	#1	#2	#1	#2	#1	#2
HROG02	2010	2004	88	48	105	97	218	105
HROG05	1205	1245	23	66	0,16	0,93	144	86
HROG06	490	575	88	95	3	1	88	88
HROG17	39	15	85	57	0,92	0,37	151	133
HROG36	1201	1235	223	159	0,79	0,42	159	184

Calculated IC_50_ values for all cell line pairs and substances tested are listed.

In summary, we did not observe any obvious discrepancy in drug sensitivity of the cell line pairs. Thus, functional drug response measurements of tumor samples obtained from individual GBM patients are not influenced by a transient cryo-preservation step before the start of culture.

## Discussion

In this study, we could show that tumor tissue from GBM surgical resection specimens can be cryo-preserved prior to successful *in vitro* culture establishment. As a technically very simple method, cryopreservation of GBM tumor tissue prior to model establishment as reported here may be quite appealing to both clinical and basic researchers alike for the following reasons:

(1) The methodology is easy and as successful as for the cell cultures established from fresh tumor material. Even though success rates tend to be lower after vital freezing than with tumor tissue fresh from surgery, this difference did not reach statistical significance (p = 1.000). This finding is in line with similar analyses of gastrointestinal tumors [15], [16] as well as for established GBM xenografts and cancer-initiating cells [17], [18]. In seven cases we observed no tumor outgrowth. In four of these cases, the tissue was captured from surgery for recurrent glioblastoma. Besides the comparable little tumor mass in these patients, the tumors were heavily pretreated according to standard therapy often causing necrotic tissue. (2) There were no clear-cut differences observed neither in morphology and growth kinetic nor in the sensitivity towards the tested drugs. Moreover, the mutational patterns of the original tumors were maintained in the cell lines in all cases. The latter may of course not withstand whole genome sequence analysis. It is, however, very likely that slight differences will also be observed when analyzing several micro samples originating from the same GBM case as has been shown for methylation patterns and the levels of receptor amplification in different sub-clones [19], [20]. (3) No obvious bias in success was observed concerning the pathomolecular profile or the clinical stage in this comparative model establishment analysis. Due to the limited number of cases analyzed this may of course change when analyzing models of many individual tumors. (4) Finally and most important, the cryo-method will allow pre-selection of interesting cases before model establishment according to molecular data of the original tumor, clinical course, therapy response or development of resistance.

Taken together, we conclude that this simple cryo-step does not interfere with successful establishment of ultra-low passage GBM cell lines or primary cultures. Ultra-low passage primary cell cultures are considered superior to continuous cell lines in brain tumor research [21]. However, their availability can be limiting to scientific progress. Thus, separating the pure collection of clinical GBM specimen from the more complex logistics of model establishment will simplify the successful generation of individual GBM models. Our data also imply that responses towards drugs were very similar between the cell line pairs and thus the procedure will also be suited for clinical response prediction of individual patients. Moreover, this will be an especially valuable technology for clinical studies. For instance, it opens up the possibility for selective model establishment of responders vs. non-responders and thus enables detailed functional tests at a later time point.

Lastly, not only pre-selection of interesting cases prior to laborious cell line establishing processes is possible, but the technique also enables repetitive establishment procedures and therefore allows going “back” to primary cultures. Of course, this will depend on the amount of GBM tissue stored immediately after operation. One simple problem is the costs for generating many cryo-aliquots of a tissue sample. This problem is obvious for academics but most likely not so in the context of clinical studies. The procedure does also not necessarily interfere with pathological analysis of the operated GBM, since cryopreserved tissue pieces can be substitute of fresh material at least for diagnostic pathological procedures. Another interesting aspect is provided by the data of Table 3: they allow the intriguing conclusion that repetitive culture generation can lead to a higher overall success rate.

The only obvious difference we observed between material originating from the clinical GBM specimen and the established cell line pairs was a loss of EGFR gene amplification. This is a well-described phenomenon and due to a loss of extra-chromosomal copies of the gene [19], [22], [23]. Since this is a culture-related process, there was no difference in this regard between the cell line pairs.

Recently, we published a similar analysis on the success of xenograft establishment of colorectal carcinomas with and without a cryopreservation step. In that analysis, we focused on xenografts since for colorectal carcinomas, they are much more likely successful than primary cell lines [15]. However, for GBM, it is just the other way around. Cell lines of GBM are much easier to generate than xenografts. Not only the engraftment of tumor pieces or biopsies is very time-consuming (2–11 months until initial engraftment) but also it generally takes 3 regraftment steps (8–18 months) until the xenograft acquires a GBM-like phenotype [24].

Last but not least, tumor cell cultures provide an unlimited source of material not only for preclinical analyses but potentially also for individual vaccines basing on autologous antigen-presenting cells and tumor antigens. In the last decade, this methodology not only reached but even surpassed standard clinical procedures [25], [26].

## Supporting Information

Figure S1 **Neuronal origin.** The expressions of GFAP, nestin and vimentin are depicted in a bar chart (mean percent expressing cells and standard deviation) with the results of the pairs displayed side by side (#1 in black and #2 in grey shading).
(TIF)

Figure S2 **Drug response.** One representative drug response curve is presented for each cell line pair and each substance. The values obtained from vitality staining were normalized (1 equaling the control of untreated cells and 0 equaling the control of dead cells). All experiments were performed in triplicates and repeated at least three times. A: response to TMZ (concentration range: 2 mM–128 nM); B: response to BCNU (concentration range: 500 µM–32 nM); C: response to Vincristine (concentration range: 244 nM–300 pM); D: response to Imatinib (concentration range: 250 µM–60 nM).
(TIF)
