# Supplementary figures and images for: Establishment and Characterization of Primary Glioblastoma Cell Lines from Fresh and Frozen Material: A Detailed Comparison
Source: PLoS One. 2013 Aug 7;8(8):e71070. doi: 10.1371/journal.pone.0071070 (PMC3737284; doi:10.1371/journal.pone.0071070)

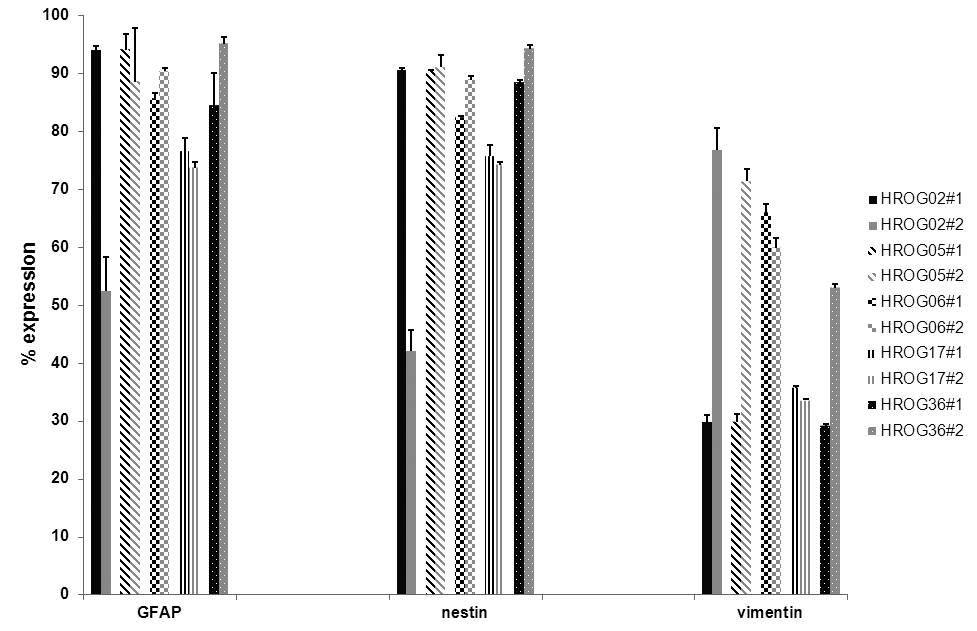

Supplement: Figure S1 — Neuronal origin. The expressions of GFAP, nestin and vimentin are depicted in a bar chart (mean percent expressing cells and standard deviation) with the results of the pairs displayed side by side (#1 in black and #2 in grey shading). (TIF) [file pone.0071070.s001.tif]

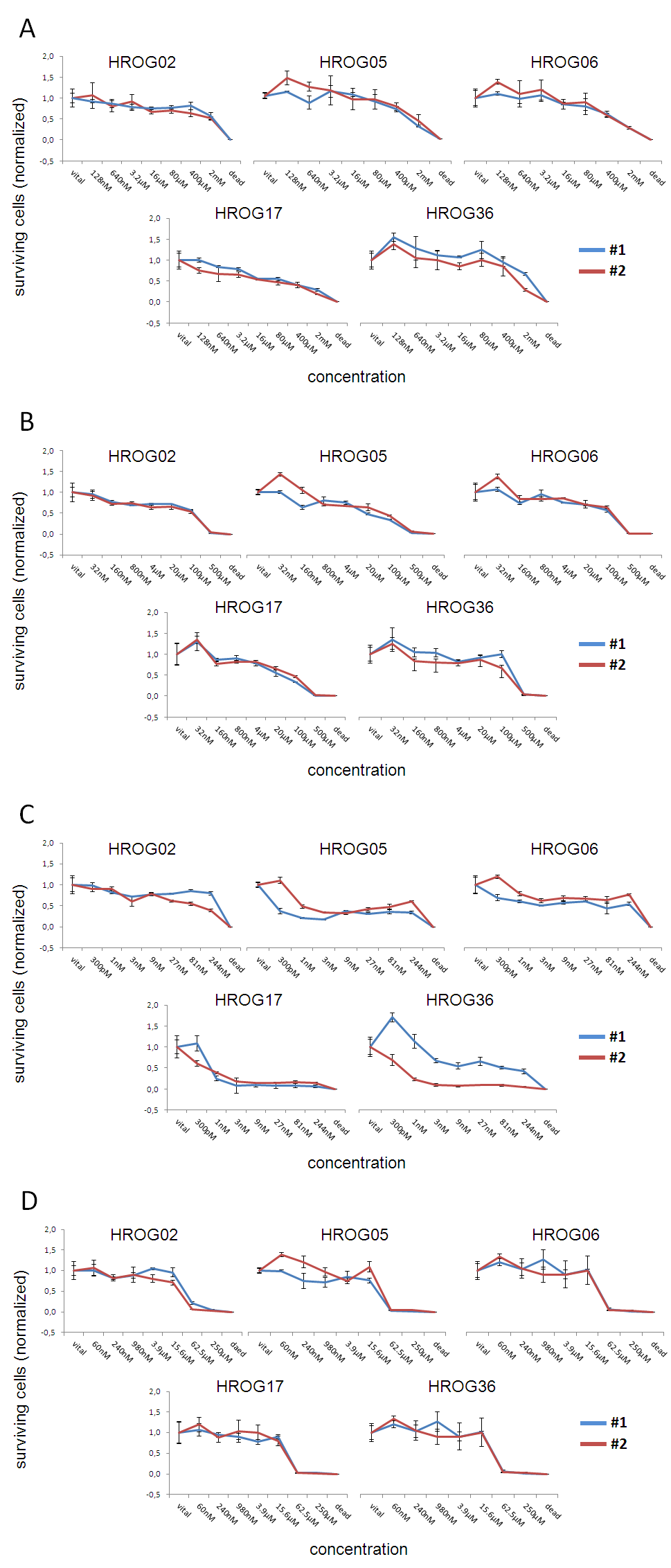

Supplement: Figure S2 — Drug response. One representative drug response curve is presented for each cell line pair and each substance. The values obtained from vitality staining were normalized (1 equaling the control of untreated cells and 0 equaling the control of dead cells). All experiments were performed in triplicates and repeated at least three times. A: response to TMZ (concentration range: 2 mM–128 nM); B: response to BCNU (concentration range: 500 µM–32 nM); C: response to Vincristine (concentration range: 244 nM–300 pM); D: response to Imatinib (concentration range: 250 µM–60 nM). (TIF) [file pone.0071070.s002.tif]
